# Work conflict: Another trigger to smartphone addiction of individuals with high rumination?

**DOI:** 10.1371/journal.pone.0287669

**Published:** 2023-11-13

**Authors:** Yanwei Sun, Xing Cai, Ting Nie

**Affiliations:** 1 School of Humanities, Zhuhai City Polytechnic, Zhuhai, Guangdong, China; 2 School of Finance and Insurance, Guangxi University of Finance and Economics, Guangxi, China; 3 School of Business, Macau University of Science and Technology, Macau, China; University of Sharjah, UNITED ARAB EMIRATES

## Abstract

With the widespread use of smartphones, many people spend much time on smartphones for shopping, learning, socializing, and so on, which can affect an individual’s mental health and work performance. Especially, individual perceived conflict at work may increase their social anxiety and thus raise the risk of their smartphone addiction. This study collected data from 577 corporate employees in China through convenience sampling to explain the influence mechanism of work conflict on smartphone addiction and to verify the moderating role of rumination. Statistical results show that relationship conflicts, task conflicts, and process conflicts positively affect smartphone addiction by enhancing social anxiety. Moreover, rumination positively moderates the relationship between work conflict and smartphone addiction. People with high rumination are more likely to escape reality due to conflict at work, which further enhances their smartphone addiction behaviors. Our study suggests that a relatively harmonious working atmosphere should be established within organizations, especially for employees with rumination. Work conflict is a predisposing factor for social anxiety and smartphone addiction in individuals with high rumination.

## Introduction

According to the 48th “Statistical Report on China’s Internet Development Status” released by the China Internet Network Information Center (CNNIC) in June 2022, the number of Chinese Internet users reached 1.051 billion with an increase of 19.19 million by the end of 2021, and the Internet penetration rate was 74.4% by September 2021. The number of mobile Internet users in China reached 1.007 billion, and the proportion of Internet users using smartphones to access the Internet was as high as 99.6%, which was much higher than the proportion using desktop computers, laptop computers, and tablet computers to surf the Internet [1]. Smartphones have fully penetrated all aspects of people’s lives, which has brought infinite convenience to individuals’ social interaction, work, study, shopping, and entertainment [2]. However, with the development of artificial intelligence technology, smartphones have become more than just tools to meet people’s basic daily needs. Some people also develop a fascination with the phone itself, its functions, and mobile web activities [3]. The addictive reaction is manifested as a compulsive desire to use a mobile phone. Once an individual cannot meet the needs of using a mobile phone immediately, the addict will have a series of psychological and physiological discomforts [4]. Smartphone addiction shows compulsive behaviors, high-frequency screen switching, inattention, sensitivity to mobile phone content, and even auditory hallucinations [5]. Many studies have reported the harm of smartphone addiction on adolescent growth. Parenting style, peer relations, and academic stress can predict excessive use of cell phones [6]. However, current research has paid significantly less attention to smartphone addiction in work settings, which will lead to a negative impact on employee work relations and job performance. Further clarification is needed to explain the underlying mechanisms that lead employees to become addicted to their smartphones.

Work conflict occurs frequently in the workplace, where individuals experience negative emotions due to disagreement on work processes, work tasks, or work philosophies [7]. Work conflict is a threat to the harmonious work relationship. Unfavorable living and working environments can increase perceived loneliness, and enhance the individual’s psychological stress and anxiety, which may create a tendency to escape from reality. Many people may try to obtain inner satisfaction and harmony in the virtual world, and the frequency of using cell phones increases significantly as a result. Particularly for individuals with higher rumination, who often reconsider and interpret the experienced events negatively. They tend to perceive higher levels of frustration and helplessness [8]. Thus the effect of work conflict on individual smartphone dependence may be more pronounced in groups with high rumination.

The negative effects of smartphone addiction are well recognized in academic research as well as in practice, and excessive cell phone use by employees can lead to reduced work engagement and lower performance in the workplace [9–11]. As the digital nature of work intensifies, companies need to be more focused on workplace deviance or addictive behaviors and pinpoint the high-risk groups. However, insufficient attention has been paid in previous studies to the antecedents of workplace addiction in the workplace. There are significant differences in the causes and outcomes of excessive smartphone use between adults and adolescents. This research attempts to explain a possible cause of smartphone addiction from the perspective of work conflict, which will further enrich the research on the formation mechanism of smartphone addiction. We also hope to raise the attention of organizations and managers to build a harmonious working relationship and atmosphere. By reducing the likelihood of excessive smartphone use, companies can better fulfill their corporate social responsibility. Through statistical investigation, we try to examine the mediating role of social anxiety and the moderating role of rumination between work conflict and smartphone addiction. Based on a review of the related literature, this study proposed a theoretical model and validated the hypotheses by analyzing data from 577 employees, and finally theoretical and practical implications were discussed. It is hoped that managers can take positive interventions to reduce the occurrence of smartphone addiction and its negative effects in the workplace.

## Theoretical basis and research hypotheses

### Work conflict and smartphone addiction

Work conflict is a dynamic process that occurs between interdependent parties, in which negative emotions are reflected when the two parties’ perceived disagreement and the realization of the goals are blocked [12]. Therefore, work conflict will happen when interacting individuals are inconsistent with their needs, interests, goals, and opinions or temporarily unable to coordinate, resulting in emotional and behavioral hostility and tension with each other. Thus, a discordant and even struggling relationship is formed [13]. There are three types of conflicts in the workplace according to their nature: relationship conflict, task conflict, and process conflict [7]. Relationship conflict refers to the conflict between people due to the inconsistency of emotions and ideas, which is accompanied by psychological hostility, tension, anger, and other negative reactions [14]; Task conflict refers to the conflict between the two parties due to differences in work views, understandings or judgments [15]; Process conflict refers to the dispute between the viewpoints and opinions of the team members on how to proceed and complete the task [7]. Work conflicts are usually destructive. They could undermine work synergy, threaten mental health, waste resources, reduce team cohesion, and increase employee hostility and aggressiveness [16, 17]. They might also enhance employee perception of boredom and reduce creativity, which leads to higher turnover and absenteeism [18]. Reduced productivity and satisfaction are also related to dissonance in the workplace [19, 20]. Teams with lower conflict tend to exhibit higher cohesion and teamwork with a greater willingness to learn within the team, and they are more likely to demonstrate higher team performance [21].

Smartphone addiction is usually defined by five aspects: tolerance, withdrawal, craving, avoidance of other problems, and negative consequences [22]. It is a phenomenon in which an individual could no longer control his use of a mobile phone after excessive and inappropriate contact with a smartphone, which leads to an addiction tendency and psychological or behavioral changes [23]. Although smartphones could improve people’s work efficiency and quality of life, they might also have a negative impact on our physical and mental health, such as depression, anxiety, loneliness, and other negative emotional problems [24–26]. If an individual has a high degree of smartphone dependence, it might cause changes in physical functions such as pain and weakness, and psychological changes such as decreased concentration [27]. Once the use of smartphones is banned, smartphone addicts might experience anxiety, irritability, a desire to use the mobile phones immediately, and an inability to focus on the current task [28, 29]. Smartphone addiction also may cause a certain degree of damage to an individual’s attention, memory ability, delayed gratification ability, time management ability, and brain areas [30, 31]. Excessive use of smartphones could also negatively affect work performance and relationships with family, friends, classmates, and teachers [32].

Work conflict increases the individual’s cognitive load and interferes with effective cognitive processes, thereby affecting efficacy, creativity, and decision-making [33]. Work environments with high conflict are often accompanied by complex interpersonal relationships and inconsistent working styles. They can increase employees’ workload and burnout, which may easily lead to a conscious reduction in their work engagement and even to high turnover behaviors [34, 35]. Research on adolescents has confirmed that smartphone addiction is closely related to individual families and interpersonal conflicts, which is an important factor that is not conducive to youth growth [36, 37]. Smartphone addiction is more pronounced when individuals feel unkindness or aggression in the environment [38]. When people perceive a disharmonious environment at work and cannot agree with their colleagues on work philosophy, methods, or processes, they may feel unable to fit into the team, resulting in a high level of loneliness. Social isolation is one of the factors that trigger mobile phone addiction [39]. Smartphone use increases as many people divert their attention to avoid exacerbating conflict. They will be more likely to be immersed in the virtual world of the internet. Therefore, we propose research hypothesis 1.

Hypothesis 1.1: Relationship conflict has a positive influence on employee smartphone addiction.

Hypothesis 1.2: Task conflict has a positive influence on employee smartphone addiction.

Hypothesis 1.3: Process conflict has a positive influence on employee smartphone addiction.

### The mediating effect of social anxiety

In the process of social development, if individuals’ social role and social status are very different from the goals they set, they will have anxiety [40]. Social anxiety is defined as the negative emotions an individual perceived in social interaction situations, which will lead to a certain degree of pain and functional impairment [41]. It is not caused by the individual’s insufficient mastery of skills, but by the individual’s evaluation of self-expression and self-concept in social interaction [42]. When individuals experience low self-esteem, low self-concept, low self-efficacy, low self-evaluation, or low self-attention, they are more prone to anxiety [43, 44]. Therefore, social anxiety comes from individual negative expectations about the evaluation of real or imagined social situations.

Social anxiety is not conducive to the individual’s social activities and the development of harmonious interpersonal relationships [45]. Relationship conflicts tend to increase individual anxiety, reduce the ability to solve problems collaboratively, and thereby reduce performance [46]. Task conflicts increase personal stress perception and negatively evaluate one’s own abilities [47]. The process conflicts caused by task delegation or role assignment imply the evaluation or respect of the individual’s ability. Individuals who disagree with the task assignment might feel that the task is lower than their ability, and think that the task assignment is an insult [48]. Conflict at work can easily disrupt cohesion within the organization, which is a threat to the harmonious work atmosphere and tends to create higher levels of psychological stress and anxiety among employees [49]. Individuals with higher social anxiety usually have lower self-perceived emotion-regulating efficacy and more criticism of themselves [50]. The fear of not having a cell phone is mainly due to social anxiety about missing out and falling behind [51]. Fear of missing out and interaction anxiety have been proven to be effective in predicting the time individuals spend on mobile phones [52]. Individuals with higher social anxiety generally feel unsafe, and more sensitive to interpersonal relationships. Therefore, they will use smartphones more often to establish contact with others [53]. In the internet world, social needs and recognition needs are more easily met, which can alleviate the anxiety caused by conflicts in reality. As a result, individuals with higher social anxiety are more likely to show mobile phone dependence [54, 55]. Therefore, work conflict can increase individual social anxiety and lead to individual smartphone addiction. We propose research hypothesis 2.

Hypothesis 2.1: Social Anxiety mediates the relationship between relationship conflict and employee smartphone addiction.

Hypothesis 2.2: Social Anxiety mediates the relationship between task conflict and employee smartphone addiction.

Hypothesis 2.3: Social Anxiety mediates the relationship between process conflict and employee smartphone addiction.

### The moderating effect of rumination

Individuals who encounter significant challenges or frustrations are prone to pessimistic thinking, which leads them to focus on themselves too much and neglect communication with the outside world. Even after the negative event, the individual is still immersed in negative emotions and can not get out. This way of thinking is defined as rumination [56]. Rumination originates from the difference between reality and perceived goals, and it is a state of conscious self-management. After experiencing negative life events, people will repeatedly think about the causes and consequences of negative emotions, which in turn awakens previous negative memories. They may negatively interpret the current situation, create a sense of helplessness and frustration, and dampen the individual’s confidence in problem-solving [8]. Rumination directly affects individual negative emotions such as social anxiety, depression, and loneliness [57], which tends to affect the quality of sleep and enhance psychological stress and may even increase the risk of suicide [58, 59]. Individuals with high rumination tend to have more negative behaviors such as aggressive behaviors and withdrawal behaviors [60]. They usually show lower levels of self-esteem and well-being in the workplace, which are also more destructive to maintaining favorable interpersonal relationships in teams [61]. The more frustration and adversity an individual perceives in daily life, the more likely he or she is to ruminate about it, which results in more negative emotional and behavioral reactions [62]. When individuals encounter unfair treatment in the workplace, rumination can induce a large number of deviant behaviors [63, 64].

Goal conflicts and interpersonal dissonance at work will be enlarged by the individual’s rumination. Individuals tend to repeatedly think about the stressful event and their anxiety will increase [65, 66]. Due to the presence of false emotions and cognitive biases, people may seek out cell phones to gain relief or avoid negative emotions. High rumination tends to lead individuals to magnify the negative effects of a work conflict. Therefore, some employees may use their phones more often to escape the dissonance and inconsistency in reality. Therefore, we propose research hypothesis 3.

Hypothesis 3.1: Rumination moderates the positive relationship between relationship conflict and employee smartphone addiction, in such a sense that higher levels of rumination strengthen the positive relationship.

Hypothesis 3.2: Rumination moderates the positive relationship between task conflict and employee smartphone addiction, in such a sense that higher levels of rumination strengthen the positive relationship.

Hypothesis 3.3: Rumination moderates the positive relationship between process conflict and employee smartphone addiction, in such a sense that higher levels of rumination strengthen the positive relationship.

**Fig 1:**
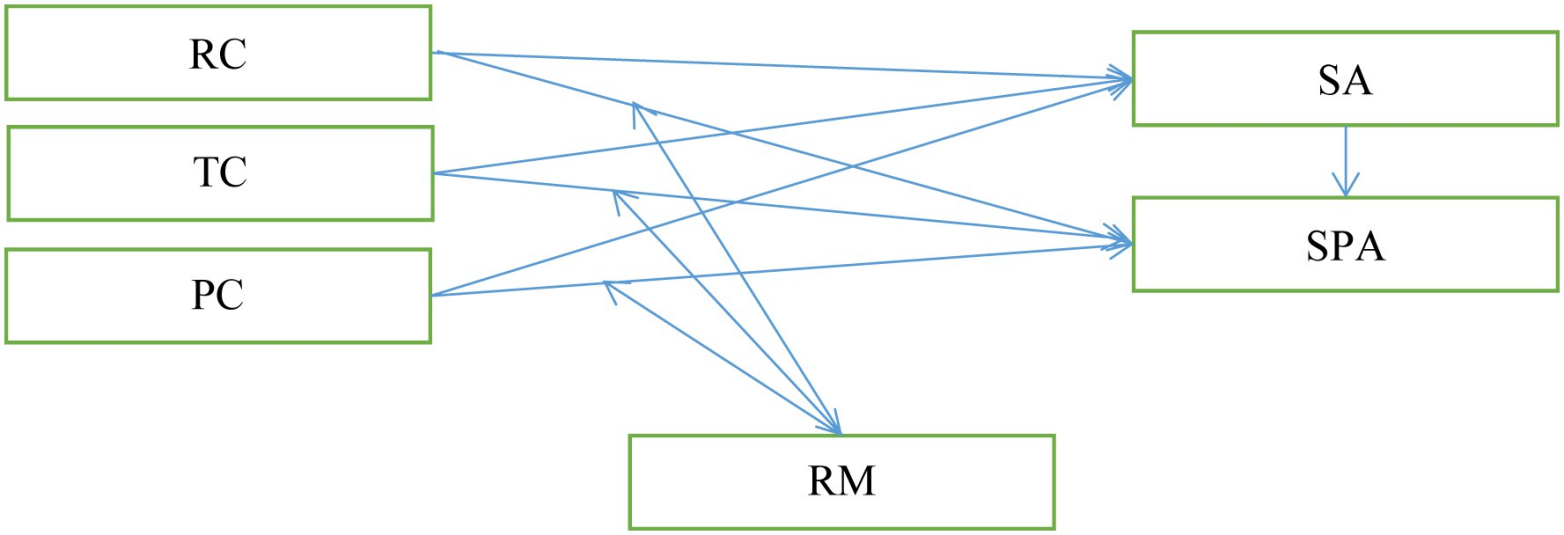
Theoretical model. Notes: RC: Relationship Conflict; TC: Task Conflict; PC: Process Conflict; SA: Social Anxiety; SPA: Smartphone Addiction; RT: Rumination.

The Theoretical Model for this study is shown in Fig 1.

## Method

### Procedure

Nowadays, 99.6% of Chinese people use their cell phones to get convenience in society. Smartphones have penetrated everyone’s life in all aspects [1]. With the development of the Internet and communication technologies, this trend will become increasingly apparent in many countries. While smartphones bring benefits to people, they can also cause many problems due to overuse. The study based on the Chinese sample also has some implications for other countries.

In order to examine the impact of work conflict on smartphone addiction, we collected employee data from Guangdong, Guangxi, Shanxi, Hunan, Hubei, Beijing, Chongqing, Macau, and other domestic provinces or cities in China through questionnaires. The study was approved by the IRB of Zhuhai City Polytechnic and written content was obtained from all the anticipates. The survey was conducted on a voluntary basis and participants were informed about the purpose of the survey. All questionnaires were anonymous and guaranteed to be used for academic research only. Respondents can choose to fill out an online questionnaire or a paper questionnaire. They may stop answering at any time, and incomplete questionnaires were considered invalid.

In this study, the data were collected through an online questionnaire at one-month interval to reduce the effect of common method bias. Time1: questionnaires were collected through convenience sampling and were used to measure work conflict; social anxiety and rumination; A total of 700 questionnaires were distributed and 642 valid questionnaires were returned, with a valid return rate of 91.7%. Time2: questionnaires were distributed to the same group of participants with matching telephone numbers to measure smartphone addiction and demographic characteristics. A total of 642 questionnaires were distributed and 577 valid questionnaires were returned, with a valid return rate of 89.9%

The data were analyzed by Amos 24, SPSS 26.0, and Process 3.4. It mainly involves reliability test, validity test, descriptive statistical analysis, correlation analysis, hierarchical regression analysis, and bootstrap test.

### Participants

Respondents in the study are all employees of Chinese companies. Among the valid samples, there are 267 men, accounting for 46.3% of the total, and 310 women, accounting for 53.7% of the total. The ratio of men and women is relatively balanced. There are 177 respondents under the age of 30, accounting for 30.7% of the total; 339 respondents between the ages of 31 and 40, accounting for 58.8% of the total, and only 48 respondents over the age of 40, accounting for 8.3% of the total. The respondents are relatively young. Most respondents have received higher education. There are 481 people with a bachelor’s degree or above, accounting for 83.4% of the total. The survey respondents involve multiple industries such as manufacturing, construction, service, information industry, etc.

### Measures

All the scales in this survey are self-reported, which was measured by Likert five-point scale (*From 1 completely disagree to 5 completely agree*).

***Work Conflict*** is defined as a dynamic process that occurs between interdependent parties. When the two parties perceive a disagreement or the realization of the goal is hindered, they reflect negative emotions [12]. The measurement of work conflict is based on the research of Jehn and Mannix (2001) [14]. There are 9 items in total with three dimensions: relationship conflict (e.g. I sometimes have some emotional conflicts with my colleagues at work), task conflict (e.g. There are sometimes opinion conflicts in the work I am responsible for), and process conflict (e.g. I sometimes have disputes with my colleagues over resource allocation at work). The internal consistency coefficients are 0.815, 0.779, and 0.851 respectively.

***Social Anxiety*** is defined as the negative emotions experienced in social interaction situations [41]. The measurement of social anxiety is based on the study of Scheier, et al.(1985) [67]. There are 6 items in total (e.g. I often feel anxious when speaking in public) and the internal consistency coefficient is 0.892.

***Smartphone Addiction*** is defined as a form of excessive and inappropriate contact with smartphones without control, which leads to psychological or behavioral changes [23]. The measurement of smartphone addiction is based on the study of Bianchi and Phillips (2005) [22]. There are 28 items in total (e.g. I feel lost without a cell phone) and the internal consistency coefficient is 0.914.

***Rumination*** is defined as repeated thinking about the current situation, causes, and consequences of a stressful event in life [56]. The measurement of rumination is based on the study of Treynor, et al. (2003) [68]. There are 22 items in total (e.g. I often think about my shortcomings, failures, and mistakes)and the internal consistency coefficient is 0.944.

***Control variables***: Previous studies have shown that demographic variables such as gender, age, education et. al have significant effects on individual smartphone addiction [69–71]. The impact on smartphone addiction may also vary due to the different intensity and frequency of conflict faced by employees across industries [72, 73]. Therefore, gender, age, education, and industry were considered as control variables in the study. The classification are as follows: gender (man; woman); age(less than 30; 31–40;41-50; 51–60;more than 60); education (high school; bachelor; master; PhD.); industry(manufacturing, construction, service, information industry and so on).

## Results

### Confirmatory factor analysis

First of all, to control the effect of common method bias, we used Harman’s single-factor test as well as confirmatory factor analysis [74]. In Harman’s single-factor test, the variance explained by the first factor was 25.12%, which is much below than the threshold level of 50%. Moreover, confirmatory factor analysis is used to verify the validity and test the common method bias. Table 1 shows that the model fit of the Six-factor model(Relationship Conflict; Task Conflict; Process Conflict; Social Anxiety; Smartphone Addiction; Rumination.) is the best (*χ*^2^/*df* = 2.807, GFI = 0.906, CFI = 0.949, RMSEA = 0.051, RMR = 0.035), which has reached an acceptable level and is significantly better than other alternative models. The model fit of the one-factor model is far from acceptable (*χ*^2^/*df* = 12.498, GFI = 0.620, CFI = 0.666, RMSEA = 0.129, RMR = 0.107). Therefore, the common method deviation of the data is not serious in the study.

**Table 1:** Confirmatory factor analysis(N = 577).

Model	Factors	*χ* ^2^	df	*χ*^2^/*df*	GFI	CFI	RMSEA	RMR
Six-factor model	RC,TC,PC,SA,SPA,RT	1170.714	417	2.807	0.906	0.949	0.051	0.035
Four-factor model	RC+TC+PC,SA,SPA,RT	1485.323	419	3.545	0.882	0.928	0.061	0.041
Three-factor model	RC+TC+PC,SA+SPA,RT	3082.161	423	7.286	0.757	0.820	0.105	0.096
Two-factor model	RC+TC+PC,SA+SPA+RT	3767.714	427	8.844	0.715	0.774	0.107	0.097
One-factor model	RC+TC+PC+SA+SPA+RT	5361.841	429	12.498	0.620	0.666	0.129	0.107

Table notes: RC: Relationship Conflict, TC: Task Conflict, PC: Process Conflict, SA: Social Anxiety, SPA: Smartphone Addiction, RT: Rumination.

### Correlation analysis

Correlation analysis shows when controlling gender, age, education, and industry factors, the relevant variables in the study are all significantly correlated. The results are shown in Table 2. Relationship conflict (0.341**), task conflict (0.246**), and process conflict (0.270**) are positively associated with social anxiety. Relationship conflict (0.388**), task conflict (0.305**), and process conflict (0.347**) are positively associated with smartphone addiction. Moreover, social anxiety (0.442**) is positively associated with smartphone addiction. Relationship conflict (0.321**), task conflict (0.344**), process conflict (0.293**), social anxiety (0.283**), and smartphone addiction (0.317**) are positively associated with rumination. Research hypothesis 1 has been initially verified.

**Table 2:** Correlation statistics(N = 577).

	Mean	SD	1	2	3	4	5	6	7	8	9
Gender	1.77	0.424									
Age	1.83	0.710	0.215**								
Education	2.07	0.676	-0.182**	-0.147**							
Industry	3.92	1.345	0.042	-0.033	0.023						
RC	3.00	0.650	-0.090*	-0.124**	-0.031	-0.057					
TC	3.19	0.543	-0.108**	-0.107*	0.051	0.002	0.686**				
PC	3.11	0.654	-0.118**	-0.075	0.048	-0.011	0.636**	0.688**			
SA	3.17	0.573	-0.042	-0.053	0.012	0.041	0.341**	0.246**	0.270**		
SPA	3.11	0.683	-0.053	-0.080	0.006	-0.037	0.388**	0.305**	0.347**	0.442**	
RT	3.70	0.797	-0.148**	-0.100*	-0.014	-0.040	0.321**	0.344**	0.293**	0.283**	0.317**

Table notes:

** p < 0.01,

* p < 0.05

RC: Relationship Conflict, TC: Task Conflict, PC: Process Conflict, SA: Social Anxiety, SPA: Smartphone Addiction, RT: Rumination.

### Hierarchical regression analysis

Hierarchical regression is used to verify the influence of work conflict on social anxiety and smartphone addiction [75]. The results are shown in Table 3. Models 1 to 3 verify the influence of work conflict on social anxiety. After controlling gender, age, education, and industry, relationship conflict (0.343**), task conflict (0.242**), and process conflict (0.267**) positively affect social anxiety significantly. Models 4 to 6 verify the influence of work conflict on smartphone addiction. After controlling gender, age, education, and industry, relationship conflict (0.384**), task conflict (0.299**), and process conflict (0.343**) positively affect smartphone addiction significantly. Model 7 verifies the influence of social anxiety on smartphone addiction. After controlling gender, age, education, and industry, social anxiety (0.441**) positively affects smartphone addiction significantly. Therefore, research hypotheses 1.1, 1.2, and 1.3 have been verified.

**Table 3:** Results of hierarchical regression analysis (N = 577).

	SA	SA	SA	SPA	SPA	SPA	SPA
	M1	M2	M3	M4	M5	M6	M7
Gender	-0.009	-0.014	-0.007	-0.009	0.011	-0.002	-0.022
Age	-0.003	-0.023	-0.031	-0.029	-0.049	0.058	-0.055
Education	0.019	-0.007	-0.008	0.013	-0.017	-0.018	-0.010
Industry	0.060	0.040	0.043	-0.016	-0.038	-0.034	-0.055
RC	0.343***			0.384***			
TC		0.242***			0.299***		.
PC			0.267***			0.343***	
SA			.				0.441***
△*R*^2^	0.120	0.063	0.079	0.152	0.097	0.125	0.202
F	15.612***	7.682***	9.342***	20.533***	12.253***	16.253***	28.951***

Table notes:

***p < 0.001,

** p < 0.01,

* p < 0.05

RC: Relationship Conflict, TC: Task Conflict, PC: Process Conflict, SA: Social Anxiety, SPA: Smartphone Addiction, RT: Rumination.

### Bootstrap analysis

Process 3.4 is used to verify the mediating role of social anxiety between work conflict and smartphone addiction and the moderating role of rumination between interpersonal conflict and smartphone addiction at different levels [76]. The sample size of the bootstrap method is 5000, and the test is carried out under the 95% confidence interval. The results are shown in Table 4.:

**Table 4:** Mediation and moderation (N = 577).

		Mediating Effect						Moderating Effect				
Variables		Effect	SE	*p*	95% CI			Effect	SE	*p*	95% CI	
					Lower	Upper					Lower	Upper
RC	DE	0.226	0.042	0.000	0.144	0.307	Int	0.094	0.042	0.025	0.012	0.176
	IDE	0.112	0.019	0.000	0.076	0.153	L	0.151	0.058	0.009	0.037	0.264
							H	0.301	0.048	0.000	0.206	0.396
TC	DE	0.197	0.049	0.000	0.101	0.293	Int	0.114	0.051	0.027	0.013	0.214
	IDE	0.109	0.022	0.000	0.069	0.153	L	0.106	0.066	0.105	-0.022	0.235
							H	0.287	0.062	0.000	0.166	0.409
PC	DE	0.206	0.040	0.000	0.128	0.284	Int	0.124	0.041	0.003	0.043	0.204
	IDE	0.094	0.018	0.000	0.061	0.131	L	0.108	0.054	0.045	0.002	0.213
							H	0.305	0.049	0.000	0.209	0.401

Table notes: RC: Relationship Conflict, TC: Task Conflict, PC: Process Conflict, DE: Direct Effect, IDE: Indirect Effect, L: Low, H: High, Int: Interaction

First of all, we discuss the mediating role of social anxiety between relationship conflict, task conflict, and process conflict with smartphone addiction. The 95% confidence interval of the direct influence of relationship conflict on smartphone addiction (b = 0.226, SE = 0.042) is [0.144, 0.307], and the interval does not contain zero; The 95% confidence interval of the indirect influence between relationship conflict and smartphone addiction through social anxiety(b = 0.112, SE = 0.019) is [0.076, 0.153], and the interval does not contain zero, which means social anxiety plays a mediating role between relationship conflict and smartphone addiction. The 95% confidence interval of the direct influence of task conflict on smartphone addiction (b = 0.197, SE = 0.049) is [0.101, 0.293], and the interval does not contain zero; The 95% confidence interval of the indirect influence between task conflict and smartphone addiction through social anxiety (b = 0.109, SE = 0.022) is [0.069, 0.153], and the interval does not contain zero, which means social anxiety plays a mediating role between task conflict and smartphone addiction. The 95% confidence interval of the direct influence of process conflict on smartphone addiction (b = 0.206, SE = 0.040) is [0.128, 0.284], and the interval does not contain zero; The 95% confidence interval of the indirect influence between process conflict and smartphone addiction through social anxiety(b = 0.094, SE = 0.018) is [0.061, 0.131], and the interval does not contain zero, which means social anxiety plays a mediating role between process conflict and smartphone addiction. Therefore, hypotheses 2.1, 2.2, and 2.3 have been verified.

Then we discuss the moderating effect of rumination between relationship conflict, task conflict, and process conflict with smartphone addiction. The 95% confidence interval of the interaction effect between relationship conflict and smartphone addiction (b = 0.094, SE = 0.042) is [0.012, 0.176]. The interval does not contain zero and the moderating effect exist. At a low level of rumination(Mean-SD), the 95% confidence interval of the relation between relationship conflict and smartphone addiction (b = 0.151, SE = 0.058) is [0.037, 0.264], and the interval does not contain zero. While at a high level of rumination(Mean+SD), the 95% confidence interval of the relation between relationship conflict and smartphone addiction (b = 0.301, SE = 0.048) is [0.206, 0.396], and the interval does not contain zero. The direct influence of relationship conflict on smartphone addiction exists at both low and high levels of rumination. A high level of rumination strengthens the positive influence of relationship conflict on smartphone addiction. The 95% confidence interval of the interaction effect between task conflict and smartphone addiction (b = 0.114, SE = 0.051) is [0.013, 0.214]. The interval does not contain zero and the moderating effect exist. At a low level of rumination(Mean-SD), the 95% confidence interval of the relation between task conflict and smartphone addiction (b = 0.106, SE = 0.066) is [-0.022, 0.235], and the interval does contain zero, which means the direct influence of task conflict on smartphone addiction does not exist at a low level of rumination. While at a high level of rumination(Mean+SD), the 95% confidence interval of the relation between task conflict and smartphone addiction (b = 0.287, SE = 0.062) is [0.166, 0.409], and the interval does not contain zero. The direct influence of task conflict on smartphone addiction only exists at a high level of rumination. A high level of rumination strengthens the positive influence of task conflict on smartphone addiction. The 95% confidence interval of the interaction effect between process conflict and smartphone addiction (b = 0.124, SE = 0.041) is [0.043, 0.204]. The interval does not contain zero and the moderating effect exist. At a low level of rumination(Mean-SD), the 95% confidence interval of the relation between process conflict and smartphone addiction (b = 0.108, SE = 0.054) is [0.002, 0.213], and the interval does not contain zero. While at a high level of rumination(Mean+SD), the 95% confidence interval of the relation between process conflict and smartphone addiction (b = 0.305, SE = 0.049) is [0.209, 0.401], and the interval does not contain zero. The direct influence of process conflict on smartphone addiction exists at both low and high levels of rumination. A high level of rumination strengthens the positive influence of process conflict on smartphone addiction. Therefore, research hypotheses 3.1, 3.2, and 3.3 have been verified.

## Discussions

The increasing popularity of smartphones has brought great convenience to people’s work and life, but also caused some negative psychological and physiological effects [25, 26]. In the past two decades, a large number of studies have verified the existence of smartphone addiction among adolescents. Their academic performance, social interactions, and mental health are all affected [77, 78]. Relatively few studies have focused on smartphone addiction among adults in the workplace. In fact, mobile phone addiction has more negative effects on adults, such as lower performance, family conflicts, and physical and psychological harm, which will also bring huge losses to their family and organizations [79, 80]. The reasons why adults are addicted to mobile phones are very different from those of young people. The influence of the original family is weakened, and the situational factor at work may be more prominent [81]. This study attempted to explain possible causes of smartphone addiction from the perspective of a perceived work conflict.

Through a statistical survey, the study explored the influence mechanism of work conflict on smartphone addiction through social anxiety in the workplace and the moderating role of rumination between work conflict and social addiction. On the basis of statistical analysis, relationship conflict, task conflict, and process conflict all positively affect smartphone addiction. When people encounter higher relationship conflict, task conflict, and process conflict at work, their tendency toward smartphone addiction will increase. Social anxiety plays a mediating role in the relationship between relationship conflict, task conflict, process conflict, and smartphone addiction. Work conflict can increase individual social anxiety and increase the occurrence of smartphone addiction. This is consistent with findings in previous studies on adolescents that when individuals perceive rejection and alienation from peers, both psychological stress and anxiety rise as a result, and they are more likely to overuse the internet [82]. The findings of this study further confirm that disharmonious relationships in the workplace are also contributing factors to smartphone addiction. Even if it is due to inconsistency in work content and work procedures, it also makes individuals use cell phones more and increases the risk of smartphone addiction.

Previous studies have verified some moderating effects under the formation mechanism of mobile phone addiction, such as demographic characteristics, mindfulness, belonging, trust, etc. [83–86]. Whereas rumination as a negative trait has received extensive attention in trauma research, individuals with high rumination have a tendency to think automatically and repeatedly after negative experiences including recurring thoughts about the cause, effect, and meaning of negative emotions and events at the time [56]. Rumination increases the risk of psychiatric disorders such as depression, anxiety, obsessive-compulsive disorder, and post-traumatic stress disorder, and individuals with rumination have more difficulty recovering from traumatic experiences [87]. The present study has further confirmed the negative effect of rumination in the working scenario. Rumination positively moderates the relationship between work conflict and smartphone addiction. Individuals with high rumination are more likely to develop avoidance thinking due to work conflicts encountered at work, which will enhance their smartphone addiction behaviors.

## Conclusions

### Theoretical implications

The influence of smartphones has penetrated all aspects of our lives and work. In this survey, most of the participants stated that they used smartphones for three to five hours a day. Some even stated that they used smartphones for more than 10 hours a day, which was far more than the average Chinese use of mobile phones: 100.57 minutes per day (2020). When mobile phones cannot be used, individuals will feel a strong sense of insecurity and become extremely unaccustomed. Work efficiency and life satisfaction will decrease as a result. When an individual’s over-reliance on smartphones seriously affects the individual’s life and work, it is an unhealthy behavior like all addictive tendencies. The negative outcomes of smartphone addiction have been well documented, and the antecedents of smartphone addiction deserve to be explored in depth in order to inhibit its negative effects more effectively. Unlike previous studies, the causes of cell smartphone addiction were explained from the perspective of individual traits and family environment. This study confirms that conflict at work is also a determinant of smartphone addiction, especially for employees with ruminative thinking. Negative experiences at work often lead to destructive behaviors, which may turn the originally harmonious interpersonal relationship into conflict. Work conflict will induce individual anxiety, which will affect their normal social activities and life so that they will spend more time immersed in the illusory world of mobile phone networks. This is particularly the case for individuals who are prone to pessimistic thoughts when encountering negative events and repeatedly think about the reasons and results. They are more likely to escape reality due to conflicts and thus spend much time on smartphones. Individuals with rumination may enhance the influence of interpersonal conflict on smartphone addiction. If the excessive use of smartphones reaches the state of addiction, it will cause damage to the individual’s emotions, body, and mental status, and induce unpredictable negative behaviors.

### Managerial implications

Conflicts in the workplace are almost inevitable. From the individual perspective, as a cause of smartphone addiction, employees should strive to maintain a positive and optimistic attitude when facing such conflict and cope with the current difficulties, rather than passively avoiding them. Individuals should change their way of thinking, establish developmental thinking, and solve problems proactively. A negative mindset easily fuels individual smartphone addiction. From the organizational perspective, companies should strive to create a harmonious working atmosphere and reduce possible work conflicts. Companies can avoid employee addiction to smartphones by encouraging cooperation, stress management, team building, and so on. Organizations need to pay attention to the occupational health of employees. Managers should try to match employees with their positions through job design, help employees formulate career plans, provide employees with vocational training, give employees certain autonomy in decision-making, and minimize the possibility of conflicts at work. Organizations should try to create a safe and healthy working environment to reduce employees’ perceived anxiety in the workplace and improve occupational well-being.

### Limitations and future study

There are certain research limitations in this study. First, the study used self-reported scales, which are prone to common source bias. Although two phases data collection and Harman’s single-factor test partially controlled the bias [74], the results of the study were still may be affected to some extent. In future studies, paired surveys can be considered to collect data to test research hypotheses accurately. Second, the sample in this study all came from Chinese companies. China is a collectivist country where individuals prefer to maintain good interpersonal relationships and avoid possible conflicts [88]. Employees are more likely to react negatively to perceived conflict in the workplace. This differs significantly from many countries, so there may be some external validity issues with this study. Future research could focus on cross-cultural comparisons to more accurately identify the impact of work conflict on individual smartphone addiction. Finally, with the widespread use of smartphones, the dependency and excessive use of smartphones will also cause more problems, especially for adults, which will seriously affect their work productivity and family well-being [10, 79]. This study only verified that work conflict in the workplace can enhance individuals’ social anxiety and smartphone addiction behaviors. This trend is more pronounced for individuals with high rumination. The formation of smartphone addiction is a complex process that requires in-depth explorations of possible antecedents and consequences, such as environmental factors, work characteristics, leadership style, organizational climate, personal traits, and so on. In future studies, we will also consider different research methods to further investigate the possible impact mechanisms and propose effective intervention strategies for organizations.

## Supporting information

S1 Data (SAV)
